# A retrospective cohort study of the effect of SARS-CoV-2 point of care rapid RT-PCR at the Emergency Department on targeted admission

**DOI:** 10.1186/s12879-022-07497-x

**Published:** 2022-06-13

**Authors:** Susanne E. Mortazavi, Malin Inghammar, Claus Christiansen, Anne-Katrine Pesola, Mikael Stenkilsson, Magnus Paulsson

**Affiliations:** 1 Division of Clinical Chemistry and Pharmacology, Department of Laboratory Medicine, Skåne University Hospital, Lund University, Lund, Sweden; 2 Section for Infection Medicine, Department of Clinical Sciences Lund, Skåne University Hospital, Lund University, Lund, Sweden; 3 Clinical Microbiology, Laboratory Medicine Skåne, Region Skåne, Lund, Sweden; 4 Department of Emergency and Internal Medicine, Skåne University Hospital, Lund, Sweden

**Keywords:** SARS-CoV-2, Targeted admission, Infection control, Point-of-care testing, Intrahospital transfers

## Abstract

**Background:**

To prevent nosocomial transmission of SARS-CoV-2, infection prevention control (IPC) measures are implemented for patients with symptoms compatible with COVID-19 until reliable test results are available. This delays admission to the most appropriate ward based on the medical condition. SARS-CoV-2 rapid antigen detection (RAD) tests and point-of-care (POC) rapid RT-PCR (VitaPCR) were introduced at emergency department (ED) at Skåne University Hospital, Sweden in late 2020, but the consequence on patient flow and targeted admission is unknown.

**Methods:**

Patients presenting at the emergency department of a referral hospital (N = 2940) between 13-Nov-2020 and 12-Jan-2021 were included. The study period was delimited into three periods by the introduction of RAD tests and the VitaPCR. Participant data was collected from hospital records, and outcome variables were Length-of-Stay (LoS), intrahospital transfers and targeted admission to COVID-19 ward.

**Results:**

Compared to baseline (RT-PCR only), RAD tests reduced ED Length-of-Stay (LoS) for participants with positive tests. Negative VitaPCR results reduced mean hospital LoS by 1.5 (95% CI 0.3–2.7) days and admissions to COVID-19 wards from 34.5 (95% CI 28.9–40.5) to 14.7 (95% CI 11.1–19.1) per 100 admissions and reduced transfers between hospital wards in the first 5 days from 50.0 (95% CI 45.0–55.0) to 34.0 (95% CI 30.3–37.9) per 100 admissions.

**Conclusion:**

RAD tests enabled prompt detection of SARS-CoV-2 infection which had pronounced effects on LoS at the ED. Negative VitaPCR enabled cessation of IPC measures and a negative test was associated with increased targeted admissions, reduced intrahospital transfers and shorter LoS at the hospital.

**Supplementary Information:**

The online version contains supplementary material available at 10.1186/s12879-022-07497-x.

## Background

The coronavirus disease 2019 (COVID-19) pandemic is caused by the Severe Acute Respiratory Syndrome Coronavirus 2 (SARS-CoV-2) that emerged in China in late 2019 [1]. According to WHO, on 7 November 2021 over 248,467,363 global cases and 5,027,183 global deaths have been verified [2]. Rapid detection and isolation of infected individuals are important to limit the spread of the virus and to protect patients and health care workers [3]. Real-time reverse transcription polymerase chain reaction (RT-PCR) is the gold standard for SARS-CoV-2 detection, due to high sensitivity and specificity compared to other diagnostic methods [4]. However, RT-PCR is time consuming and requires specialized laboratory settings, personnel, and instruments. As a less expensive and faster point-of-care test method, SARS-CoV-2 rapid antigen detection (RAD) tests became widely available during the autumn of 2020. However, RAD tests are generally inferior to RT-PCR in terms of sensitivity and specificity which is particularly important when testing asymptomatic patients with low pretest probability and the main objective is to rule out infection [4–6].

In late 2020, the optimized point-of-care (POC) RT-PCR VitaPCR SARS-CoV-2 Assay (Credo Diagnostics Biomedical, Singapore) was introduced and implemented at the Skåne University Hospital, Lund, Sweden. The assay utilizes a single tube for collection of the nasopharyngeal swab, cell lysis and nucleotide extraction. The total analysis time is about 20 min. Sample preparation does not require specialized laboratory setting and the reported sensitivity and specificity for SARS-CoV-2 is 99.3% and 94.7%, respectively [7, 8]. This should be compared to the RAD Clinitest Rapid COVID-19 Antigen Test (Siemens Healthineers, Erlangen, Germany) that had a reported sensitivity of 86.5% and a specificity of 99.3% when the Food and Drug Administration (FDA) granted Emergency Use Authorization (EUA). For both RAD tests and RT-PCR-based tests, the accuracy is likely to be lower in samples with low viral load.

During 2020, substantial reorganizations were made at Emergency Departments (ED) and hospital wards globally to cope with the extraordinary requirements caused by the SARS-CoV-2 pandemic, including infection prevention and control (IPC) precautions to prevent secondary cases among patients and hospital staff. The study region was largely affected by the second wave of the pandemic with a considerable increase of COVID-19 cases from 1 Sept 2020 to 31 January 2021 [9]. Due to the broad clinical manifestations of COVID-19, infection cannot be safely excluded based on clinical symptoms and signs only [10]. Before introduction of RAD tests and VitaPCR, all patients with suspected COVID-19 infection were isolated at the ED or admitted to hospital wards with IPC facilities dedicated to patients with positive or unknown COVID-19 status until RT-PCR results from the core hospital facility were available. The typical time from sampling to results ranged between 12 and 24 h. In the high prevalence setting during the second wave of the COVID-19 pandemic, a large proportion of the patients at the ED met the definition of suspected COVID-19 and were admitted to COVID-19 isolation wards instead of targeted admission to wards specialized on the true medical problem. If the COVID-19 RT-PCR test was negative, IPC precautions were discontinued, and the patient transferred to another hospital ward for continued treatment.

Although the sensitivity and specificity of the VitaPCR is superior to that of any RAD test, and time to result is substantially shorter for VitaPCR than for RT-PCR tests analyzed at the core hospital laboratory, the effects of these improvements on patient care are unknown. We hypothesized that introduction of the faster tests in the algorithm facilitated clinical decisions at the ED, limited IPC precautions to when necessary and improved targeted admission. This study evaluates the introduction of RAD tests and VitaPCR based on length-of-stay at the ED and hospital ward, intrahospital transfers the first 5 days and targeted admissions to COVID-19 ward during the peak of the second wave.

## Methods

### Study setting and design

**Fig. 1:**
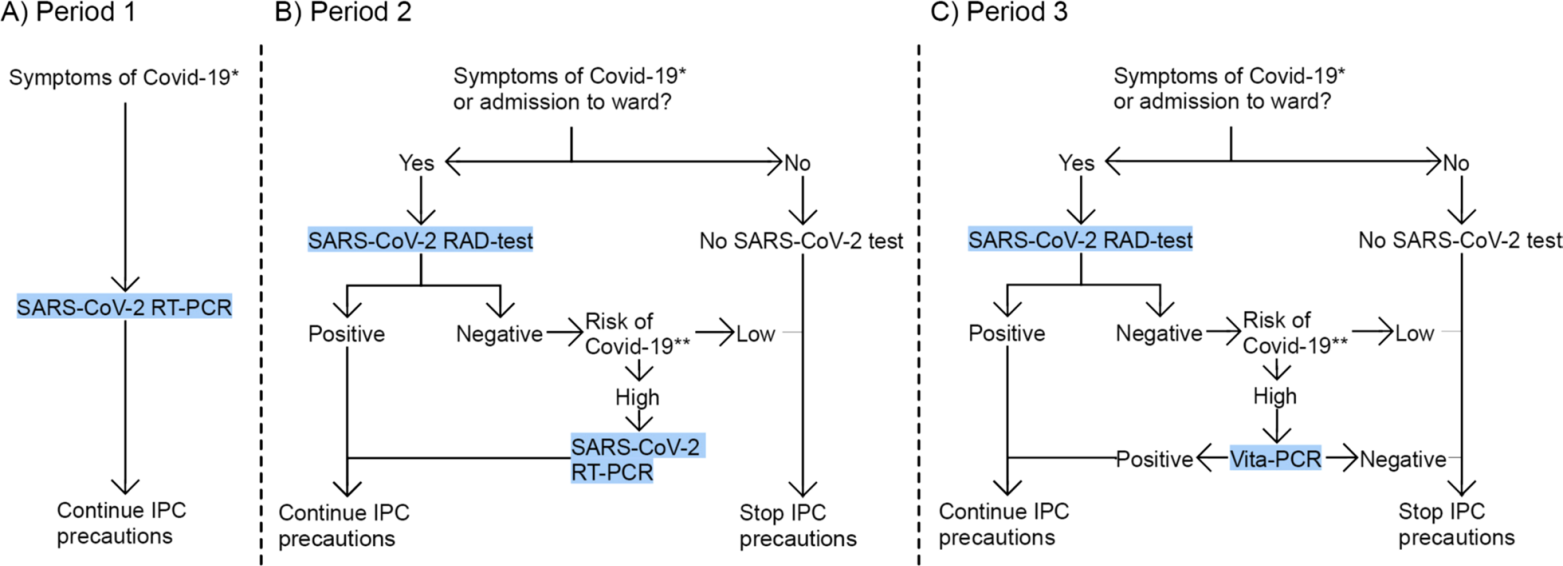
Algorithms for SARS-CoV-2 testing during the study Period 1, Period 2 and Period 3. At the Emergency Department during Period 1 (**A**), patients with symptoms of COVID-19 were tested with RT-PCR and Infection prevention and control (IPC) precautions continued. During Period 2 (**B**), a rapid antigen detection (RAD) test for SARS-CoV-2 was added, which enabled diagnosis of SARS-CoV-2 infection or discontinuation of IPC precautions for patients with low risk of SARS-CoV-2 infection and negative test result. During Period 3 (**C**), the rapid POC RT-PCR Vita-PCR was introduced, and COVID-19 specific IPC precautions were ended for Vita-PCR negative patients regardless of symptoms. *Newly developed upper or lower respiratory tract symptoms, fever, nausea, diarrhoea, malaise, anosmia or recent close contact with a person with confirmed SARS-CoV-2 infection. **Anosmia, fever with unknown origin, dyspnoea with unknown origin, cough or other respiratory symptoms and known contact with a COVID-19 case previous 7 days

This retrospective observational study is based on data from patients presenting at the Emergency Department, Skåne University Hospital, Lund, Sweden between Nov 13, 2020, and Jan 12, 2021. The hospital is a regional referral center, but the ED primarily serves the population of Lund and near surroundings (population of about 300,000). The total annual ED visits in 2020 were 59,000 patients. The present study was divided into three distinct time periods separated by the dates for introduction of RAD tests and VitaPCR, respectively: Period 1 (Nov 13–Dec 2), Period 2 (Dec 3–Dec 22) and Period 3 (Dec 23–Jan 12). The standard of care from the beginning of the pandemic and Period 1 of the present study was analysis of nasopharyngeal samples for SARS-CoV-2 with RT-PCR at a core hospital laboratory facility (Laboratory medicine Skåne, Region Skåne, Lund, Sweden). On Dec 3, 2020 (Period 2 of this study), POC rapid SARS-CoV-2 antigen detection test (ClinitestRT; Siemens Healthineers) was introduced together with an algorithm to select which analysis method that was to be used. On Dec 23, POC testing with VitaPCR was added to the algorithm. The algorithms are presented in Fig. 1. The standard of care for suspect or confirmed patients with COVID-19 were unchanged during the study period and no changes were made concerning routines for hospital admissions. The average weekly COVID-19 incidence rate in Skåne county per 100,000 inhabitants was 355 in Period 1, 623 in Period 2 and 630 in Period 3.

### Data sources

Data on visits to the ED and hospital ward admissions were collected from the hospital records using key word search, as specified below.

### Participants

The hospital records for all adult patients (≥ 18 years) that presented at the ED during the study period were screened for inclusion in the study. Inclusion criterions were any of the following: known COVID-19 infection at presentation to the ED, treatment in isolation room in the ED, emergency alerts labeled “Infection”, or emergency alerts with main complaints marked as “dyspnea”, “fever”, “infection”, “confusion”, “shock”, “cardiac arrest” and “non-specified illness”. All eligible patients during the study period were included.

### Variables

SARS-CoV-2 test results and test methods were recorded and used for grouping of participants into independent groups: “Positive test at the ED” and “Negative test at the ED” included participants for which a nasopharynx test was taken at the ED and analyzed for SARS-CoV-2 by RT-PCR, RAD test or VitaPCR. For RAD test and VitaPCR, the test result was available before the patient left the ED, while this was not the case for RT-PCR. Participants with positive SARS-CoV-2 analyzed before presentation to the ED were not sampled for SARS-CoV-2 and here labelled as “Positive test before admission”. Finally, “Not tested” included participants not tested for SARS-CoV-2 at the ED and the medical records did not specify COVID-19 status. The ICD-10 diagnoses at discharge from the ED and hospital wards were recorded and aggregated into compound variables (Additional file 1). Data on admission and transfer between wards were recorded and wards were grouped in the compound variables “COVID-19 ward” which were designated wards for patients with suspect or diagnosed COVID-19, “Mixed COVID-19/Internal Medicine ward” (wards with ICP facilities but not dedicated exclusively for patients with COVID-19), “Intensive care unit” (ICU) or “Other”, which included a broad range of non-COVID-19 hospital wards. For each subject, sex and age were recorded and presented together with diagnoses as descriptive data. In all analyses, study period 1–3 were considered for exposure variables. Outcome variables were ED Length-of-Stay (LoS), discharge to home from ED, admission to hospital ward, hospital LoS, intrahospital transfers and targeted admissions. LoS were calculated for each participant from the time of arrival to the ED until the time of discharge from the ED or hospital ward.

### Statistical analyses

Continuous variables with normal distribution are expressed as mean ± standard deviation (SD) or 95% confidence intervals (CI). Categorical variables are presented as counts, fractions of total and 95% CI calculated with the Wilson/Brown method to allow for comparisons between groups (overlapping ranges represent insignificant differences). Comparisons were also evaluated by One-Way-ANOVA with Tukey’s multiple comparison tests and by Fisher’s exact test. Analyses were performed with GraphPad Prism 9.0 (GraphPad Software, San Diego, CA, USA). P-values < 0.05 were considered statistically significant. Missing data is presented in the tables.

## Results

### Study cohort and participant enrollment

A total of 9325 patients visited the Emergency Department during the study period and 2940 of these met the inclusion criteria and were selected for enrollment in the study. There was a consecutive increase in the total number of patients that visited the ED and an increase in the proportion that met the inclusion criteria: In Period 1: 781 participants out of 3024 patient visits (25.8%) met the criteria, in Period 2: 988 participants out of 3149 patient visits (31.4%), and Period 3: 1171 participants out of 3152 patient visits (37.2%). The mean age was 60.8 (SD ± 20.8) years and 1497 (50.9%) of the participants were women.

### SARS-CoV-19 testing and test results

During the study period, a total of 1866 (63.5%) participants were tested for COVID-19 at the ED. There was no significant difference in testing percentage between men and women (48.3% vs 51.7%). The mean age among participants that were tested for SARS-CoV-2 was 64.1 (SD ± 20.0) years and the mean age among those not tested was 55.1 (SD ± 21.0) years. As the study periods were defined by changes in testing routines, substantial differences in SARS-CoV-2 testing methods were observed between the periods (Table 1). Samples were analyzed with more than one method for 318 of the 2940 participants (10.8%). The most common combination was RAD and RT-PCR (n = 186, 6.3%), followed by RAD and VitaPCR (n = 75, 2.6%), which is consistent with the testing algorithms in use during Period 2 respectively Period 3 for patients with negative RAD test but high risk of COVID-19 (Fig. 1). After introduction of the RAD test and VitaPCR, there was a significant decrease in RT-PCR test analyses. In Period 3, RT-PCR was used in 9.5% (n = 111) of the 1171 participants. In most cases this was used in combination with one of the other testing methods, and as the only test in 2.0% (n = 24) in Period 3 (Table 1).

**Table 1:** SARS-CoV-2 analysis methods used at the Emergency department

	Period 1 (N = 781)		Period 2 (N = 988)		Period 3 (N = 1171)		Total (N = 2940)	
	n =	% (95% CI)	n =	% (95% CI)	n =	% (95% CI)	n =	% (95% CI)
Analysis method								
RT-PCR	443	56.7 (53.2–60.2)	298	30.2 (27.4–33.1)	111	9.5 (7.9–11.3)	852	29.0 (27.4–30.6)
RAD test	1	0.1 (0.007–0.7)	506	51.2 (48.1–54.3)	395	33.7 (31.1–36.5)	902	30.7 (29.0–32.4)
VitaPCR	0	0.0 (0.0–0.5)	4	0.4 (0.2–1.0)	435	37.1 (34.4–40.0)	439	14.9 (13.7–16.3)
Total	444	56.9 (53.4–60.3)	650	65.8 (62.8–68.7)	772	65.9 (63.2–68.6)	1866	63.5 (61.7–65.2)
Participants tested with multiple methods								
Antigen and RT-PCR	0	0.0 (0.0–0.5)	152	15.4 (13.3–17.8)	34	2.9 (2.1–4.0)	186	6.3 (5.5–7.3)
Antigen and Vita-PCR	0	0.0 (0.0–0.5)	0	0.0 (0.0–0.4)	75	6.4 (5.1–8.0)	75	2.6 (2.0–3.2)
VitaPCR and RT-PCR	0	0.0 (0.0–0.5)	2	0.2 (0.04–0.7)	44	3.8 (2.8–5.0)	46	1.6 (1.2–2.1)
RAD, VitaPCR and RT-PCR	0	0.0 (0.0–0.5)	2	0.2 (0.04–0.7)	9	0.8 (0.4–1.5)	11	0.4 (0.2–0.7)

The use of Real-time polymerase chain reaction (RT-PCR) at the core laboratory decreased significantly between each study period as the point of care rapid antigen detection (RAD) test and point of care rapid RT-PCR VitaPCR were introduced in Period 2 and 3 respectively. Number of tests are presented with percentages (%) of total participants in each period and 95% confidence intervals (CI)

Of the 2940 participants, 408 (13.9%) tested positive for SARS-CoV-2 infection at the ED, 1458 (49.6%) tested negative, 568 (19.0%) had had a positive test before admission to the ED, and 506 (17.2%) were not tested for SARS-CoV-2 infection at the ED (Table 2). The fraction of participants that were not tested with any method before admission to or at the ED decreased significantly during the study period, from 31.1% in Period 1, to 14.7% in Period 2 and 9.9% in Period 3 (Table 2). At the ED, 1866 participants were tested, some of these with multiple testing methods. The total amount of tests taken at the ED was 2193 and of these 449 were positive (20.5%). The proportion of positive tests increased significantly during the study period (Period 1: 70 positive of 443 tested participants, 15.8%, 95% CI 12.7–19.4%; Period 2: 156 positive of 808 tested, 19.3%, 95% CI 16.7–22.2%; Period 3: 222 positive of 941 tested, 23.6%, 95% CI 21.0–26.4%).

**Table 2:** Characteristics of all study participants at the Emergency Department

	Period 1 (N = 781)		Period 2 (N = 988)		Period 3 (N = 1171)		P value	Total (N = 2940)	
	n =	% (95% CI)	n =	% (95% CI)	n =	% (95% CI)		n =	% (95% CI)
Demographic data of study participants									
Age [mean (± SD)]	61 (± 22)		66 (± 18)		61 (± 20)		P = 0.04	61 (± 21)	
Female sex	417	46.6 (43.1–50.1)	489	50.5 (47.4–53.6)	591	49.5 (46.6–52.3)	P = 0.27	1497	50.9 (49.1–52.7)
SARS-CoV-2 test results									
Positive test at the ED	70	9.0 (7.2–11.2)	135	13.7 (11.7–15.9)	203	17.3 (15.3–19.6)		408	13.9 (12.7–15.2)
Negative test at the ED	374	47.9 (44.4–51.4)	515	52.1 (49.0–55.2)	569	48.6 (45.7–51.5)		1458	49.6 (47.8–51.4)
Positive test before admission	92	11.8 (9.7–14.2)	193	19.5 (17.2–22.1)	283	24.2 (21.8–26.7)		568	19.3 (17.9–20.8)
Not tested	245	31.4 (28.2–34.7)	145	14.7 (12.6–17.0)	116	9.9 (8.3–11.8)		506	17.2 (15.9–18.6)
ICD-10 diagnosis at discharge from ED									
Covid-19	53	6.8 (5.2–8.85)	146	14.8 (12.7–17.1)	229	19.6 (17.4–21.9)		428	14.6 (13.3–15.9)
Respiratory symptoms	109	14.0 (11.7–16.6)	163	16.5 (14.3–18.9)	143	12.2 (10.5–14.2)		415	14.1 (12.9–15.4)
Other infection	103	13.3 (11.0–15.7)	107	10.8 (9.0–12. 9)	106	9.1 (7.5–10.8)		316	10.7 (9.7–11.9)
Chest pain or heart disease	95	12.2 (10.1–14.6)	95	9.6 (7.9–11.6)	107	9.1 (7.6–10.9)		297	10.1 (9.1–11.2)
Abdominal pain or GI symptoms	93	11.9 (9.8–14.4)	78	7.9 (6.4–9.7)	102	8.7 (7.2–10.5)		273	9.3 (8.3–10.4)
Other internal medicine	58	7.4 (5.8–9.5)	54	5.5 (4.2–7.1)	78	6.77 (5.4–8.2)		190	6.5 (5.6–7.4)
Neurological deficit or symptoms	53	6.4 (5.2–8.8)	48	4.9 (3.7–6.4)	59	5.0 (3.9–6.4)		160	5.4 (4.7–6.3)
Other respiratory tract infection	35	4.5 (3.2–6.2)	30	3.0 (2.1–4.3)	41	3.5 (2.6–4.7)		106	3.6 (3.0–4.3)
Trauma	25	3.2 (2.2–4.7)	34	3.4 (2.5–4.8)	29	2.5 (1.7–3.5)		88	3.0 (2.4–3.7)
Other orthopedic diagnose	20	2.6 (1.7–3.9)	16	1.6 (1.0–2.6)	34	2.9 (2.1–4.0)		70	2.4 (1.9–3.0)
Other surgery or urology	20	2.6 (1.7–3.9)	10	1.0 (1.9–0.6)	17	1.5 (0.9–2.3)		47	1.6 (1.2–2.1)
No diagnosis	91	11.7 (9.6–14.1)	173	17.5 (15.3–20.0)	180	15.4 (13.4–17.6)		444	15.1 (13.9–16.4)
Other	26	3.3 (2.3–4.8)	34	3.4 (2.5–4.8)	46	3.9 (3.0–5.2)		106	3.6 (3.0–4.3)
Discharge from ED									
Discharge to home from ED, total	395	50.6 (47.1–54.1)	478	48.4 (45.3–51.5)	580	49.5 (46.7–52.4)		1453	49.4 (47.6–51.2)
Positive test at the ED	20	2.6 (1.7–3.9)	50	5.1 (3.9–6.6)	81	6.9 (5.6–8.5)		151	5.1 (4.4–6.0)
Negative test at the ED	119	15.2 (12.9–17.9)	207	21.0 (18.5–23.6)	268	22.9 (20.6–25.4)		594	20.2 (18.8–21.7)
Positive test before admission	51	6.5 (5.0–8.5)	104	10.5 (8.8–12.6)	136	11.6 (9.9–13.6)		291	9.9 (8.9–11.0)
Not tested	205	26.2 (23.3–29.4)	117	11.8 (10.0–14.0)	95	8.1 (6.7–9.8)		417	14.2 (13.0–15.5)
Hospital admissions, total	386	49.4 (45.9–52.9)	510	51.6 (48.5–54.7)	591	50.5 (47.6–53.3)		1487	50.6 (48.8–52.4)
Positive test at the ED	50	6.4 (4.9–8.3)	85	8.6 (7.0–10.5)	122	10.4 (8.8–12.3)		257	8.7 (7.8–9.8)
Negative test at the ED	255	32.7 (29.5–36.0)	308	31.2 (28.4–34.1)	301	25.7 (23.3–28.3)		864	29.4 (27.8–31.1)
Positive test before admission	41	5.2 (3.9–7.0)	89	9.0 (7.4–11.0)	147	12.6 (10.8–14.6)		277	9.4 (8.4–10.5)
Not tested	40	5.1 (3.8–6.9)	28	3.6 (2.5–5.1)	21	1.8 (1.2–2.7)		89	3.0 (2.5–3.7)
ICD-10 diagnosis, death at ED or hospital ward									
Covid-19	13	1.7 (1.0–2.8)	29	2.9 (2.1–4.2)	58	5.0 (3.9–6.3)		100	3.4 (2.8–4.1)
Other infection	3	0.4 (0.1–1.1)	11	1.1 (0.6–2.0)	11	0.9 (0.5–1.7)		25	0.9 (0.6–1.3)
Cardiovascular disease	15	1.9 (1.2–3.1)	6	0.6 (0.3–1.3)	17	1.5 (0.9–2.3)		38	1.3 (0.9–1.8)
Neurological disease	7	0.9 (0.4–1.8)	2	0.2 (0.04–0.7)	9	0.8 (0.4–1.5)		18	0.6 (0.4–1.0)
Malignancy	3	0.4 (0.1–1.1)	5	0.5 (0.2–1.2)	3	0.3 (0.1–0.8)		11	0.4 (0.2–0.7)
Other	3	0.4 (0.1–1.1)	8	0.8 (0.4–1.6)	1	0.1 (0.004–0.5)		12	0.4 (0.2–0.7)
Hospital deaths, total	44	5.6 (4.2–7.5)	61	6.2 (4.8–7.9)	99	8.5 (7.0–10.2)		204	6.9 (6.1–7.9)

Demographic data, SARS-CoV-2 test results and discharge diagnoses of all included patients, in all three study periods. The fraction of participants with positive SARS-CoV-2 tests taken at the Emergency department (ED) or before admission to the ED increased during the study period, as did Covid-19 diagnosis at discharge. ICD-10 diagnoses were grouped into compound variables (described in detail in Additional file 1). Total number of participants in each period (N =) and in each subgroup (n =) are presented with percentage as fraction of total and 95% confidence interval (CI) to enable comparisons between the periods, or mean value with standard deviation (SD), as indicated. P-values calculated by One-Way-ANOVA with Tukey’s multiple comparison tests

### Emergency Department Length-of-Stay

The mean Length-of-Stay (LoS) at the ED was 374 (SD ± 269) minutes (Table 3). This did not change significantly between the periods, but we observed a significant reduction in ED LoS between the three periods for participants with “Positive test at the ED” (P = 0.0002) or “Not tested” participants (P < 0.0001). Mean ED LoS for participants with “Positive test at the ED” decreased by 28 min (95% CI 3.0–53.0) (P = 0.02) after introduction of RAD test, and another 15 min (95% CI − 7.6 to 37.6) after introduction of the VitaPCR. Participants that were “Not tested” for SARS-CoV-2 at the ED had a LoS at the ED that was reduced by 102 min (95% CI 76.3–127.7) after introduction of the RAD test (P < 0.0001), which further decreased slightly [10 min; (95% CI − 33.16 to 13.16)] in Period 3.

**Table 3:** Length-of-Stay at Emergency Department and hospital wards

Emergency Department Length-of-Stay [mean min (± SD)]	Period 1 (N = 781)	Period 2 (N = 988)	Period 3 (N = 1171)	P value	Total (N = 2940)
All study participants	383 (± 263)	377 (± 271)	363 (± 263)	0.22	374 (± 269)
Positive test at the ED	393 (± 237)	365 (± 209)	350 (± 225)	0.0002	362 (± 222)
Negative test at the ED	430 (± 276)	442 (± 307)	423 (± 278)	0.31	431 (± 288)
Positive test before admission	296 (± 219)	313 (± 201)	297 (± 228)	0.15	302 (± 218)
Not tested	345 (± 251)	243 (± 183)	253 (± 247)	< 0.0001	295 (± 237)

Hospital Length of Stay [mean days (± SD)]	Period 1 (N = 386)	Period 2 (N = 510)	Period 3 (N = 591)	P value	Total (N = 1487)
Positive test at the ED	8.5 (± 7.4)	9.3 (± 9.1)	8.0 (± 11)	0.08	8.6 (± 11.0)
Negative test at the ED	6.6 (± 8.2)	5.8 (± 6.9)	5.1 (± 8)	0.01	5.6 (± 8)
Positive test before admission	1.4 (± 3.0)	2.7 (± 5.8)	5.9 (± 34.8)	0.004	3.0 (± 17.3)
Not tested	8.2 (± 29.1)	6.1 (± 12.5)	5.8 (± 9.2)	0.09	6.7 (± 12.7)
All participants	5.0 (± 11.9)	6.0 (± 8.8)	6.7 (± 14.7)	0.1	6.1 (± 12.2)

Length-of-stay (LoS) in each study period at the Emergency department (ED) and Hospital. Numbers indicate mean minutes and standard deviation (SD) for the ED LoS and mean days and SD for hospital LoS. P-value calculated by Fisher’s exact test

### Emergency department discharge to home and hospital wards

COVID-19 diagnoses at discharge from the ED increased significantly during the three study periods, from 6.8% in Period 1 (95% CI 5.2–8.85%), to 14.8% (95% CI 12.7–17.1) in Period 2 and 19.6% (95% CI 17.4–21.9) in Period 3. No statistically significant changes were seen for other diagnoses.

Of the 2940 participants, 1487 (50.6%) were admitted to a hospital ward and 1453 (49.4%) discharged to home. For the entire study population, the fraction of participants that were discharged to home from the ED did not change notably during the study period. However, a larger fraction of the participants that were tested for SARS-CoV-2 could be discharged to home from the ED after introduction of RAD tests and VitaPCR (Table 2, Discharge to home).

The fraction of participants that were admitted to a hospital ward did not change significantly between the study periods (Table 2, Hospital admissions). However, after introduction of the VitaPCR in Period 3 admissions of participants with “Negative test at the ED” was significantly reduced. In addition, the fraction of untested participants that were admitted was also decreased.

### Hospital admissions and Length-of-Stay

The mean LoS during the entire study period was 6.1 (SD ± 11.0) days (Table 3). The mean LoS in Period 1 was 5.0 days, increased to 6.0 days in Period 2 and 6.7 days in Period 3 (P = 0.11). However, there was a significant reduction in mean hospital LoS between the three periods for participants who had a “Negative test at the ED”. Following introduction of the RAD test in Period 2, LoS was reduced from 6.6 to 5.8 days (P = 0.27). The LoS was further reduced to 5.1 days after introduction of VitaPCR in Period 3 (Period 1 vs Period 3: P = 0.008; Period 2 vs Period 3: P = 0.046). The observed increase in hospital LoS was mainly an effect of participants that had a “Positive test before admission” to the ED for whom the mean hospital LoS increased from 1.4 (SD ± 3.0) days in Period 1 to 2.7 (SD ± 5.8) days in Period 2 and 5.9 (SD ± 34.8) (P < 0.004) days in Period 3.

### Targeted admission and intrahospital transfers

**Fig. 2:**
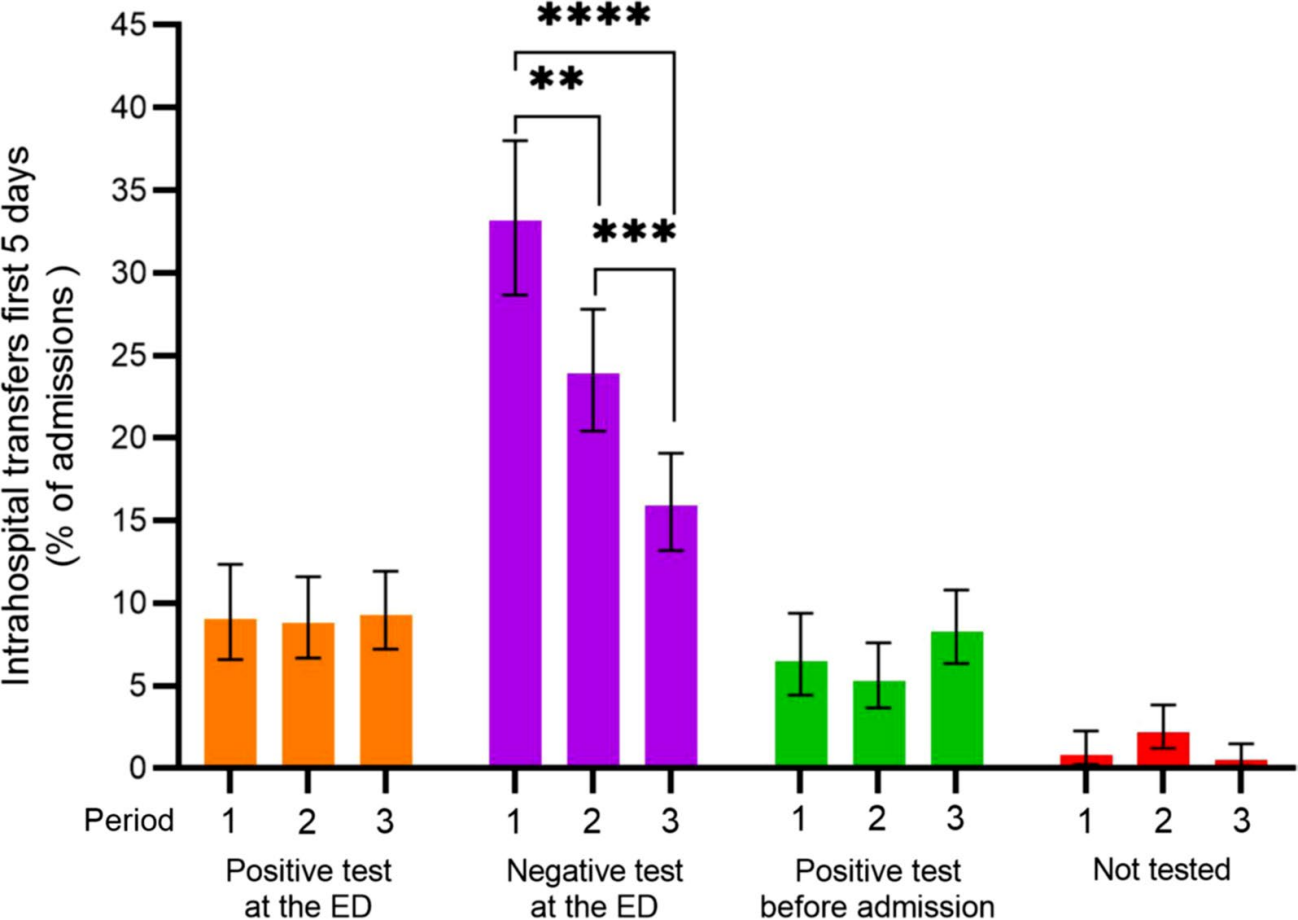
Intrahospital transfers first 5 days after hospital admission. Percentage of admitted participants that were transferred between hospital wards during the first 5 days of admission from the emergency department (ED). Participants with “Negative test at the ED” were less likely to be transferred in Period 2 and 3, after introduction of the SARS-CoV-2 rapid antigen detection (RAD) test and VitaPCR respectively. Bar shows mean value in percent and bars 95% confidence interval. **Indicate P ≤ 0.01, ***P ≤ 0.001 and ****P ≤ 0.0001, calculated with Fisher’s exact test

The overall proportions of participants that were admitted to COVID-19 wards, Mixed COVID-19/Internal medicine wards, ICU or Other wards did not vary significantly during the study period (Table 4). However, introduction of the algorithms including RAD test and VitaPCR were associated with a significant 32.0% decline in intrahospital transfers the first 5 days after admission (P < 0.0001). Hospital transfers for participants with “Negative test at the ED” decreased from 128 participants out of 386 [33.2%; 95% CI 28.7–38.0%)] in Period 1, to 122 participants out of 510 [23.9% (95% CI 20.4–27.8%)] in Period 2 and 94 participants out of 591 [15.9% (95% CI 13.2–19.1%)] in Period 3 (Fig. 2). There were no significant changes in intrahospital transfers between the periods for participants with positive tests or participants that were not tested.

**Table 4:** Admission to hospital wards and intrahospital transfers

	Period 1 (N = 386)		Period 2 (N = 510)		Period 3 (N = 591)		Total (N = 1487)	
	n =	% (95% CI)	n =	% (95% CI)	n =	% (95% CI)	n =	% (95% CI)
Admissions to ward								
Covid-19 ward	166	43 (38.2–48)	229	44.9 (40.6–49.2)	274	46.4 (42.4–50.4)	669	45 (42.5–47.5)
Mixed Covid-19/internal medicine ward	86	22.3 (18.4–26.7)	91	17.8 (14.8–21.4)	98	16.6 (13.8–19.8)	275	18.5 (29.8–34.6)
ICU	10	2.6 (1.4–4.7)	7	1.4 (0.7–2.8)	6	1 (0.5–2.2)	23	1.5 (1–2.3)
Other	115	29.8 (25.4–34.5)	166	32.5 (28.6–36.7)	197	33.3 (29.7–37.2)	478	32.1 (29.8–34.6)
Missing data	9	2.3 (1.2–4.4)	17	3.3 (2.1–5.3)	16	2.7 (1.7–4.4)	42	2.8 (2.1–3.8)
Total	386	100.0 (99.0–100.0)	510	100.0 (99.3–100.0)	591	100.0 (99.4–100.0)	1487	100.0 (99.7–100.0)
Intrahospital transfers first 5 days								
Positive test at the ED	35	9.1 (6.6–12.3)	45	8.8 (6.7–11.6)	55	9.3 (7.2–11.9)	135	9.1 (7.7–10.6)
Negative test at the ED	128	33.2 (28.7–38.0)	122	23.9 (20.4–27.8)	94	15.9 (13.2–19.1)	344	23.1 (21.1–25.3)
Positive test before admission	25	6.5 (4.4–9.4)	27	5.3 (3.7–7.6)	49	8.3 (6.3–10.8)	101	6.8 (5.6–8.2)
Not tested	3	0.8 (0.2–2.3)	11	2.2 (1.2–3.8)	3	0.5 (0.1–1.5)	17	1.1 (0.7–1.8)
Total	191	50.0 (45.0–55.0)	205	40.2 (36.0–44.5)	201	34.0 (30.3–37.9)	597	40.1 (37.7–42.7)
ICD-10 diagnosis on discharge								
Covid-19	81	21.0 (17.2–25.3)	161	31.5 (27.6–35.7)	241	40.8 (37.0–44.9)	298	32.5 (30.1–34.9)
Other infection	80	20.7 (25.0–25.0)	80	15.7 (12.8–19.1)	56	9.5 (7.4–12.1)	208	14.5 (12.8–16.4)
Chest pain or heart disease	43	11.1 (8.4–14.7)	52	10.2 (7.8–13.1)	48	8.1 (6.2–10.6)	144	9.6 (8.2–11.2)
Other respiratory tract infection	38	9.8 (7.3–13.2)	50	9.8 (7.5–12.7)	49	8.3 (6.3–10.8)	136	9.2 (7.8–10.8)
Surgery or Urology	33	8.5 (6.2–11.8)	31	6.1 (4.3–8.5)	48	8.1 (6.2–10.6)	102	7.5 (6.3–9.0)
Other internal medicine	31	8.0 (5.7–11.2)	29	5.7 (4.0–8.0)	38	6.4 (4.7–8.7)	93	6.6 (5.4–8.0)
Neurological deficit or symptoms	25	6.5 (4.4–9.4)	28	5.5 (3.8–7.8)	33	5.6 (4.0–7.8)	75	5.8 (4.7–7.1)
Respiratory symptoms	15	3.9 (2.4–6.3)	23	4.5 (3.0–6.7)	22	3.7 (2.5–5.6)	52	4.0 (3.1–5.2)
Malignancy	15	3.9 (2.4–6.3)	12	2.3 (1.3–4.1)	14	2.4 (1.4–3.9)	38	2.8 (2.0–3.7)
Trauma	7	1.8 (0.9–3.7)	13	2.5 (1.5–4.3)	11	1.9 (1.0–3.3)	26	2.1 (1.5–2.9)
Abdominal pain and GI symptoms	6	1.6 (0.7–3.3)	9	1.8 (0.9–3.3)	6	1.0 (0.5–2.2)	17	1.4 (0.9–2.1)
Other orthopedic diagnose	0	0.0 (1–0.0)	1	0.2 (0.01–1.1)	2	0.3 (0.1–1.2)	13	0.2 (0.1–0.6)
No diagnosis	9	2.3 (1.2–4.4)	11	2.2 (1.2–3.8)	12	2.0 (1.2–3.5)	30	2.2 (1.5–3.0)
Other	3	0.8 (0.2–2.3)	11	2.2 (1.2–3.8)	10	1.7 (0.9–3.1)	14	1.6 (1.1–2.4)

Intrahospital transfers between wards the first 5 days after admission from the Emergency Department (ED) decreased after introduction of SARS-CoV-2 rapid antigen detection test (Period 2) and further with the introduction of the point of care rapid RT-PCR VitaPCR (Period 3). Total number of participants in each period (N =) and in each subgroup (n =) are presented with percentage of N = and 95% confidence interval (CI), or mean value with standard deviation (SD), as indicated. P-values calculated by One-Way-ANOVA with Tukey’s multiple comparison tests

Similarly, participants with “Negative test at the ED”, were 57% less likely to be admitted to a COVID-19 ward and 81% more likely to be admitted to a non-COVID-19 hospital ward after the introduction of both the RAD test and VitaPCR (Table 5). For participants with positive tests before or at the ED, or that were not tested, no significant changes were seen during the study period.

**Table 5:** Targeted admissions to Covid-19 or other hospital wards

	Period 1		Period 2		Period 3		Total	
	n =	% (95% CI)	n =	% (95% CI)	n =	% (95% CI)	n =	% (95% CI)
Positive test at the ED (N = 257)								
All admissions	50	100 (92.9–100.0)	85	100 (95.7–100.0)	122	100 (96.9–100.0)	257	100 (98.5–100.0)
Covid-19 ward	41	82.0 (69.2–90.2)	62	72.9 (62.7–81.2)	101	82.8 (75.1–88.5)	204	79.4 (74.0–83.9)
Mixed Covid-19/internal medicine ward	6	12.0 (5.6–23.8)	17	20.0 (12.9–29.7)	17	13.9 (8.9–21.2)	40	15.6 (11.6–20.5)
ICU	1	2.0 (0.1–10.5)	2	2.4 (0.4–8.2)	1	0.8 (0.04–4.5)	4	1.6 (0.6–3.9)
Other	2	4.0 (0.7–13.5)	2	2.4 (0.4–8.2)	1	0.8 (0.04–4.5)	5	1.9 (0.8–4.5)
Missing data	0	0.0 (0.0–7.7)	2	2.4 (0.4–8.2)	2	1.6 (0.29–5.8)	4	1.6 (0.6–3.9)
Negative test at the ED (N = 864)								
All admissions	255	100 (98.5–100.0)	308	100 (98.8–100.0)	301	100 (98.7–100.0)	864	100 (99.6–100.0)
Covid-19 ward	88	34.5 (28.9–40.5)	91	29.5 (24.7–34.9)	44	14.7 (11.1–19.1)	223	25.8 (23–28.9)
Mixed Covid-19/Internal Medicine ward	68	26.7 (21.6–32.4)	57	18.5 (14.6–23.2)	63	21.0 (16.8–26.0)	188	21.8 (19.2–24.7)
ICU	9	3.5 (1.9–6.6)	4	1.3 (0.5–3.3)	5	1.7 (0.7–3.8)	18	2.1 (1.3–3.3)
Other	84	32.9 (27.5–38.9)	144	46.8 (41.3–52.3)	180	59.8 (54.2–65.2)	408	47.2 (43.9–50.6)
Missing data	6	2.4 (1.1–5)	12	3.9 (2.2–6.7)	9	3.0 (1.6–5.6)	27	3.1 (2.2–4.5)
Positive test before admission (N = 277)								
All admissions	41	100.0 (91.4–100.0)	89	100.0 (95.9–100.0)	147	100.0 (97.5–100.0)	277	100.0 (98.6–100.0)
Covid-19 ward	35	85.4 (71.6–93.1)	73	82.0 (72.8–88.6)	128	87.1 (80.7–91.6)	236	85.2 (80.5–88.9)
Mixed Covid-19/internal medicine ward	4	9.8 (3.9–22.5)	12	13.5 (7.9–22.1)	13	8.8 (5.2–14.5)	29	10.5 (7.4–14.6)
ICU	0	0.0 (0.0–8.6)	0	0.0 (0.0–4.1)	0	0.0 (0.0–2.5)	0	0.0 (0.0–1.4)
Other	1	2.4 (0.1–12.6)	2	2.2 (0.4–7.8)	2	1.4 (0.2–4.8)	5	1.8 (0.8–4.2)
Missing data	1	2.4 (0.1–12.6)	2	2.2 (0.4–7.8)	4	2.7 (1.1–6.8)	7	2.5 (1.2–5.1)
Not tested (N = 89)								
All admissions	40	100 (91.2–100.0)	28	100 (87.9–100.0)	21	100 (84.5–100.0)	89	100 (95.9–100.0)
Covid-19 ward	2	5.0 (0.9–16.5)	3	10.7 (3.7–27.2)	1	4.8 (0.2–22.7)	6	6.7 (3.1–13.9)
Mixed Covid-19/internal medicine ward	8	20.0 (10.5–34.8)	5	17.9 (35.6–7.9)	5	23.8 (10.6–45.1)	18	20.2 (13.2–29.7)
ICU	0	0.0 (0.0–8.8)	1	3.6 (0.2–17.7)	0	0.0 (0.0–15.5)	1	1.1 (0.1–6.1)
Other	28	70.0 (54.6–81.9)	18	64.3 (45.8–79.3)	14	66.7 (45.4–82.8)	60	67.4 (57.1–76.3)
Missing data	2	5.0 (0.9–16.5)	1	3.6 (0.2–17.7)	1	4.8 (0.2–22.7)	4	4.5 (1.8–11.0)

Targeted admission of patients with positive test or suspect SARS-CoV-2 infection to a COVID-19 ward or other ward with infection prevention control (IPC) facilities did not change by the introduction of rapid point of care tests (Period 2: Rapid antigen detection test; Period 3: rapid RT-PCR VitaPCR) at the Emergency Department (ED). However, test-negative patients were increasingly admitted to appropriate specialized hospital wards (“Other”) during Period 2 and 3. Total number of participants in each period (N =) and in each subgroup (n =) are presented with percentage of N = and 95% confidence interval (CI)

## Discussion

In this retrospective study, we have explored the impact of the introduction of SARS-CoV-2 RAD tests and the POC rapid RT-PCR VitaPCR on patient care during a period of high prevalence of SARS-CoV-2 infection. The study included 2940 participants that visited the ED, who were grouped into three periods to highlight differences between the previous standard of care (Core laboratory RT-PCR) in Period 1, introduction of RAD test in Period 2 and introduction of VitaPCR in Period 3. Importantly, the results reveal that the implementation had a significant effect on length-of-stay at the ED and hospital, intrahospital transfers first 5 days and targeted admission to wards with IPC facilities, which has implications for the treatment of the individual patient, patient safety, hospital infection control and optimal resource use.

Before the introduction of point-of-care rapid SARS-CoV-2 tests at the ED, patients with suspected COVID-19 infection were admitted to COVID-19 diagnostic and treatment wards with IPC facilities until results from RT-PCR became available. Patients with negative tests were then transferred to the most appropriate ward based on the medical need. While this prevented secondary cases of COVID-19, intrahospital transfers have been related to unfavorable events such as increased falls, medication errors, length-of-stay and hospital-acquired infections [11, 12], as well as increased nurse and doctor work load [13]. This study analyzed targeted admission to COVID-19 or non-COVID-19 wards from the ED, as well as using intrahospital transfers and hospital time-of-stay as surrogate markers of targeted admission. A significant improvement was seen in the outcome of all these variables that started with the introduction of the RAD test but was further pronounced after addition of the VitaPCR. According to the standard of care routines, a negative RAD test led to cessation of IPC at the ED and before admission to a hospital ward for patients without high risk of COVID-19. However, during Period 2, because of the low sensitivity of RAD-tests, it was still necessary to confirm a negative RAD test with RT-PCR at the core laboratory for patients with high risk of the infection, which in practice meant that these patients were admitted to a ward with IPC facilities and transferred to a non-COVID-19 wards only when a negative RT-PCR test was reported. In contrast, according to the algorithm used during Period 3, a negative VitaPCR was sufficient for cessation of IPC even for patients with symptoms compatible with COVID-19. Hence targeted admission was possible for negative patients regardless of symptoms. This study revealed that in Period 3, fewer patients were transferred between hospital wards, immediate initiation of appropriate therapy and shorter length-of-stay at the hospital. SARS-CoV-2 negative patients had a notable 1.5-day reduction in LoS after introduction of both tests.

No significant effect on targeted admission variables could be seen for test positive participants, likely because both suspected and confirmed cases of COVID-19 were admitted to wards with IPC measures in all study periods. However, hospital LoS for patients that had a positive SARS-CoV-2 test taken before admission to the ED was longer in Period 2 and 3 compared to the first period of the study. The reason for this is not known. No difference in age or in proportion that received ICU care (data n.s.) were seen between the periods.

High patient load at the ED puts increased pressure on hospital wards, which can lead to ED crowding, extended LoS and decreased patient safety [14]. The overall ED LoS did not substantially change during the study period, which is notable considering the increased number of patients in the latter periods of the study. However, despite that the proportion of COVID-19 diagnosis increased, participants with positive rapid tests at the ED (RAD test or VitaPCR) spent a shorter time at the ED than in Period 1. This is in line with previous results which reported a decreased time from arrival to the ED to admission at a definitive ward based on the patients COVID-19 status after introduction of POC test [15].

The fraction of patients that were discharged from the ED to home increased during the study period. This increase was seen after introduction of the RAD tests, possibly because of the faster diagnostic work up that was enabled by the rapid tests. In the algorithms used during the study period, a positive RAD test was enough to confirm COVID-19. Hence, it can be expected that any change in patient care inflicted by the rapid tests for participants that tested positive at the ED would occur already in Period 2 (after introduction of RAD test) and not change significantly after introduction of the VitaPCR.

The study period is unique in that allows for interrogation of the sequential introduction of two rapid POC analysis methods with direct implications for IPC management of patients in a high endemic setting. The study site is the only ED in the area and the hospital treats all kinds of medical emergencies, which resulted in an unbiased adult population that is generalizable to similar ED’s. The study population was sufficiently large for the proposed analysis. The conclusions drawn here may well be applicable in other similar settings, for instance the need for rapid and accurate diagnostic tools for the annual influenza virus epidemic, which put similar demands on ED and hospital wards. However, during the COVID-19 pandemic different strategies to prevent nosocomial transmission have appeared and testing strategies have evolved depending on the availability of RAD and RT-PCR tests. The effect of introducing a new test on patient flow is dependent on the setting and timing of when the test is taken. An additional test can have negative consequences on patient flow, for instance when required for transfer between hospital and nursing home [16].

Limitations of the present study mainly relate to the retrospective study design. Data collection was limited to data available in hospital records. Although the only known difference between the study periods was the testing routines, confounding factors cannot be excluded. We can only describe differences between the periods that are associated to the introduction of respective test, and not state any cause-effect. These potential biases could have been avoided with a prospective study design in which participants were randomized to RT-PCR, RAD-test or VitaPCR. At the time of introduction of these diagnostic methods, it was not possible, or ethically defendable, to conduct a prospective randomized study.

## Conclusions

In conclusion, the implementation of rapid tests for SARS-CoV-2 and an algorithm that included VitaPCR was for test-negative participants associated with an increase in targeted admissions to an appropriate ward, reduced intrahospital transfers and shortened LoS at hospital wards. For positive patients, this change was associated with shorter LoS at the ED and observed already at introduction of RAD tests. It would be of great interest to further investigate the health-economic implications of these results.

## Supplementary Information

**Additional file 1.** Grouping of ICD-10 diagnosis on discharge from the ED and hospital wards into compound variables.

## Data Availability

The datasets used and/or analyzed during the current study are available from the corresponding author on reasonable request.

## Abbreviations

RAD: Rapid Diagnostic Tests
POC: Point-of-care
ED: Emergency Department
LoS: Length-of-Stay
COVID-19: Coronavirus disease 2019
SARS-CoV-2: Severe Acute Respiratory Syndrome Coronavirus 2
RT-PCR: Real-time reverse transcription polymerase chain reaction
IPC: Infection prevention and control
ICU: Intensive Care Unit
